# Microseismic Event Location by Considering the Influence of the Empty Area in an Excavated Tunnel

**DOI:** 10.3390/s20020574

**Published:** 2020-01-20

**Authors:** Pingan Peng, Yuanjian Jiang, Liguan Wang, Zhengxiang He

**Affiliations:** 1 School of Resources and Safety Engineering, Central South University, Changsha 410083, China; dielian8@csu.edu.cn (P.P.); liguan_wang@csu.edu.cn (L.W.); hezhengxiang@csu.edu.cn (Z.H.); 2 Digital Mine Research Center, Central South University, Changsha 410083, China

**Keywords:** heterogeneous velocity model, microseismic event location, excavated tunnel, fast marching method

## Abstract

The velocity model is a key factor that affects the accuracy of microseismic event location around tunnels. In this paper, we consider the effect of the empty area on the microseismic event location and present a 3D heterogeneous velocity model for excavated tunnels. The grid-based heterogeneous velocity model can describe a 3D arbitrarily complex velocity model, where the microseismic monitoring areas are divided into many blocks. The residual between the theoretical arrival time calculated by the fast marching method (FMM) and the observed arrival time is used to identify the block with the smallest residual. Particle swarm optimization (PSO) is used to improve the location accuracy in this block. Synthetic tests show that the accuracy of the microseismic event location based on the heterogeneous velocity model was higher than that based on the single velocity model, independent of whether an arrival time error was considered. We used the heterogeneous velocity model to locate 7 blasting events and 44 microseismic events with a good waveform quality in the Qinling No. 4 tunnel of the Yinhanjiwei project from 6 June 2017 to 13 June 2017 and compared the location results of the heterogeneous-velocity model with those of the single-velocity model. The results of this case study show that the events located by the heterogeneous velocity model were concentrated around the working face, which matched the actual conditions of the project, while the events located by the single-velocity model were scattered and far from the working face.

## 1. Introduction

During the excavation of a deeply buried tunnel, rockbursts are likely to occur as the working face advances [1]. Rockbursts can have severe consequences, such as casualties, engineering delays, and economic losses [2,3]. To avoid the impact of rockbursts, a technique to provide early warnings is sought. It is determined that many microseismic events occur before rockbursts. Therefore, microseismic monitoring technology is used in many projects to provide rockburst warnings, allowing certain measures to be taken in advance of an event. At present, this technology has achieved some success in avoiding danger from underground excavation [4,5,6,7,8,9].

The accuracy of the microseismic event location (MEL) is the key to microseismic monitoring [10,11,12] because it affects the later analysis and thus the early warnings. The traditional method of MEL is based on a single-velocity model (SVM) [13,14]. This method assumes that the wave velocity in the rock mass is isotropic. However, considerable research and experimental data have shown that the location error of the SVM is very large [3,15]. For example, Jiang et al. found that the P-wave arrival of a receiver far from the source was earlier than that of a receiver near the source in microseismic monitoring of a coal mine [16]. This phenomenon indicates that the SVM is not realistic and the MEL needs to consider the path of wave propagation. Therefore, to improve the accuracy of MEL, many researchers have conducted in-depth research on velocity models [17,18]. Crosson was the first to propose the use of the least squares method to simultaneously invert the velocity and source parameters [19]. Since then, the joint inversion of the velocity and source parameter has become a hot research topic and has been widely used in engineering applications [20,21,22,23]. Konca used joint inversion to invert measured data from the Nias–Simeulue earthquake and obtained good results, emphasizing the importance of multiple data sets in seismic rupture imaging [24]. However, the joint inversion requires many measurements for the solution to be accurate and stable. Therefore, in the absence of a large amount of measurement data, the solution will be unstable due to several unknowns in the equation of the joint inversion, such as anisotropic velocity parameters, source coordinates, and origin time, which makes obtaining an accurate location (AL) difficult. Feng et al. proposed a sectional velocity model to improve the accuracy of MEL and ensure the stability of location results in an excavated tunnel [3]. In the velocity model, the sensors on the same tunnel section are treated as a single group, and the sensors on other tunnel sections are treated as other groups. It is assumed that the velocity of the wave from the microseismic source to the same set of sensors is the same, while the velocity of the wave from the microseismic source to the different sets of sensors varies. This model is particularly useful when the orientations of the strata and the tunnel are nearly perpendicular. However, in some cases, the velocity of the same group of sensors is inconsistent, such as when the orientation of the strata is at an angle to the orientation of the tunnel or the distribution of the strata is more complicated. To generate velocity models for different geological conditions, Ma et al. proposed four different equivalent velocity models [25]. However, these equivalent models cannot produce arbitrarily complex velocity models.

Therefore, establishing a complex velocity model that is consistent with the actual engineering scenario is an important factor for improving the accuracy of MEL in a tunnel. To solve this problem, Peng proposed a mesh-based velocity model [26], which can accurately generate an arbitrarily complex 3D velocity model. In this paper, based on this model, a 3D heterogeneous velocity model (HVM) is proposed for MEL around tunnels. The research methods and innovations of this paper are as follows: First, by using a grid-based modeling method that can express an arbitrary velocity model, a velocity model in the microseismic monitoring area around the excavated tunnel was established. Then, considering the influence of the empty area after excavation on the microseismic location, we proposed the use of the fast marching method (FMM) to accurately calculate the travel time of the microseismic wave from the source to the sensor. Finally, based on the precise travel time calculated using the FMM, we used a new two-step location strategy. The first step was to use block localization (BL) to determine the approximate range of the event. The second step was to use particle swarm optimization (PSO) for AL of the event within the approximate range determined in the first step. The performance of the method was analyzed with synthetic tests and the method was successfully applied to MEL in the Qinling tunnel in China.

## 2. Methodologies

In this section, we mainly describe the research methods of this article in the following three parts. First, we detail the method of establishing an arbitrary velocity model. Second, we introduce the BL step, which determines the approximate range of the source. Third, we describe the AL step, which solves the optimal location of the source using PSO. 

### 2.1. Establishment of the Velocity Model around Tunnels

With the development of computer science, many 3D modeling software packages have been developed in different fields. These software packages use grid modeling to build complex 3D geological bodies with tetrahedrons or hexahedrons. For microseismic localization, Peng proposed a grid-based velocity model [26]. Below, we briefly introduce the construction of a grid-based velocity model for a tunnel, consisting of the following four steps.

Step 1: According to the layout of the monitoring network, the monitoring area is determined. During tunnel excavation, stress concentration occurs due to the excavation of the tunnel, which disrupts the original stress pattern. When the stress is greater than the maximum stress that the rock mass can withstand, the rock mass is destroyed, and the area near the working surface is the most vulnerable. Therefore, the area near the working face is an important monitoring area in tunnel monitoring.

Step 2: According to the geological conditions in the monitoring area, a geometric model is established. In the absence of a large amount of geological data, it is difficult to obtain a complex and accurate velocity model that is consistent with the actual project [27]. Therefore, we only consider the influence of the empty area in the tunnel after excavation on the velocity model. However, the proposed method can also be applied with a complex velocity model. The wave velocity varies among different media. In a tunnel, the empty area after excavation and the unexcavated rock mass are two distinct media. The propagation velocity of P-waves in a rock mass is generally 5500–7000 m/s. However, the propagation velocity of P-waves in air is approximately 340 m/s. In this paper, these two media are considered, and an HVM for the tunnel is established to improve the MEL accuracy.

Step 3: Monitoring area meshing is then performed. The size of the block is a key parameter that affects the accuracy of the velocity model and the travel time calculated using the FMM [28], thus affecting the accuracy of the MEL. The smaller the size of the block, the higher the location accuracy of the block. However, the computational cost increases sharply as the block size gets smaller. Therefore, the determination of the block size should consider the balance between computational accuracy and computational cost.

Step 4: A geometric model is used as a constraint to assign a velocity value to the block. In this study, the tunnel monitoring area included two different media: the empty area after the excavation and the unexcavated rock mass. A given block belonged to the empty area after excavation or to the unexcavated rock mass, and the corresponding velocity was determined after deciding which medium the block belonged to. The implementation of this method is as follows: a ray is shot from the centroid of a block, and the intersections with all of the polyhedrons are counted. If the number is odd, the point is inside the polyhedron; if it is even, the point is outside. Notably, this method is only applicable to convex polyhedra. Since the tunnel model in this study was a convex polyhedron, this method was applicable. After assigning a velocity to all the blocks, an HVM was established.

**Figure 1:**
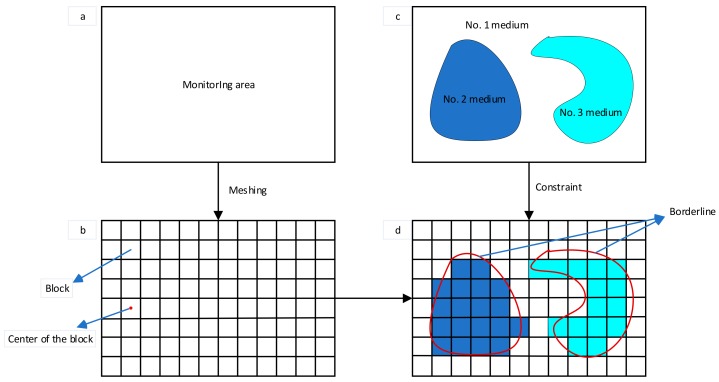
Schematic diagram of the establishment of a 2D arbitrary velocity model: (**a**) mornitoring area, (**b**) the monitoring area after meshing, (**c**) the geometry of a geological model with three media in the monitoring area, and (**d**) the figure after assigning a velocity to each block using the geometry of the geological model as a constraint.

Through the above method, the schematic diagram of the establishment of a 2D arbitrary velocity model is shown in Figure 1. Figure 1a shows the microseismic monitoring area, corresponding to step 1 above. Figure 1b shows the monitoring area after meshing, corresponding to step 3 above. Figure 1c shows the geometry of a geological model with three media in the monitoring area, corresponding to step 2 above. Figure 1d shows the figure after assigning a velocity to each block using the geometry of the geological model as a constraint, corresponding to step 4 above.

### 2.2. Block Localization

BL is used to find the block closest to the location of the source (the target block). Microseismic localization based on arrival time theory is the most widely used method [29,30,31]. The basic idea of this method is to calculate the residual between the observed arrival time and the theoretical arrival time and identify the minimum value of the residual in the space as the optimal solution. Based on dividing the monitoring area into the uniform blocks mentioned above, the center of mass of each block is adopted as the BL parameter. Each block is a potential source block. We use $\left(x_{k},y_{k},z_{k}\right)$ to represent the coordinates of the center of mass of each block, where k=1,2,3,…M and M is the total number of blocks. $\left(x_{i},y_{i},z_{i}\right)$ represents the coordinates of the i-th receiver, where i=1,2,3,…N and N is the total number of receivers. If the k−th block is the target block, the observation arrival time of the i-th receiver is:

(1) $$t_{i}^{obs}=t_{0}+T_{ik}^{obs}+\xi_{i},$$

where $t_{i}^{obs}$ is the observation arrival time of the i-th receiver, $t_{0}$ is the origin time of the source, $T_{ik}^{obs}$ is the travel time from the center of the k-th block to the i-th receiver, and $\xi_{i}$ represents the error of observation time for the i-th receiver.

Similarly, the observation arrival time of the j-th receiver is expressed as:

(2) $$t_{j}^{obs}=t_{0}+T_{jk}^{obs}+\xi_{j}.$$

By subtracting Equation (2) from Equation (1), the difference $\Delta t_{ij}^{obs}$ between the observed arrival times of the i-th and j-th receivers is obtained. $\xi_{i}$ and $\xi_{j}$ are much smaller than $T_{ik}^{obs}$ and $T_{jk}^{obs}$, respectively; therefore, we assume that $\xi_{i}$ minus $\xi_{j}$ is also equal to 0:

(3) $$\Delta t_{ij}^{obs}=T_{ik}^{obs}-T_{jk}^{obs}.$$

The corresponding theoretical arrival time difference $\Delta T_{ijk}^{rt}$ is:

(4) $$\Delta T_{ijk}^{rt}=T_{ik}^{rt}-T_{jk}^{rt}.$$

$T_{ik}^{rt}$ is the theoretical travel time from the k-th block to the i-th receiver, that is, the travel time from point $\left(x_{k},y_{k},z_{k}\right)$ to point $\left(x_{i},y_{i},z_{i}\right)$. Since the velocity model is heterogeneous, we obtain the theoretical arrival time via the FMM [28].

Therefore, the objective function is expressed as follows:

(5) $$f_{k}=\sum_{i=1}^{N-1}\sum_{j=i+1}^{N}{\left|\Delta t_{ij}^{obs}-\Delta T_{ijk}^{rt}\right|}^{m},$$

where *m* is the norm (*m* ≥ 1). One difficulty with the L2 method for acoustic emission and microseismic source location is that the input errors often do not follow a normal distribution, as is assumed by the method [32]. Therefore, we used m = 1 in this study.

According to Equation (5), the objective function value of each block is obtained, and the block with the minimum value is selected as the target block B, $f_{b}=\min(f_{1},f_{2},\ldots ,f_{M})$. The centroid coordinates of the target block are $\left(x_{b},y_{b},z_{b}\right)$.

### 2.3. Accurate Location

AL utilizes BL to find the optimal source location in the target block. The location accuracy of the block varies with the size of the block. The smaller the size of the block, the higher the location accuracy of the block. However, the computational cost increases sharply as the block size gets smaller. Therefore, in the first step of the location, we adopted an appropriate mesh size for the BL. Below, we describe the method for further location in the target block after the BL. We assume that P is any point in the target block, with coordinates of $\left(x_{p},y_{p},z_{p}\right)$, and that the velocity in the block is constant, represented by $v_{b}$. We calculate the theoretical travel time from point P to the i−th receiver using Equation (6) [26]:

(6) $$T_{ip}=\left(T_{ib}^{rt}-\left[\frac{x_{p}-x_{b}}{v_{b}}\frac{y_{p}-y_{b}}{v_{b}}\frac{z_{p}-z_{b}}{v_{b}}\right]\left[\begin{array}{l} p_{ix}^{}\\ p_{iy}^{}\\ p_{iz}^{} \end{array}\right]\right),\;x_{p},y_{p},z_{p}\subset \psi ,$$

where ψ is the spatial domain of the targeted block and ($p_{ix},p_{iy},p_{iz}$) is the gradient vector, defined as follows:

(7) $$\begin{array}{l} p_{ix}^{}=h\left(\frac{x_{i}-x_{b}}{M}\right)\\ p_{iy}^{}=h\left(\frac{y_{i}-y_{b}}{M}\right)\\ p_{iz}^{}=h\left(\frac{z_{i}-z_{b}}{M}\right) \end{array}$$

(8) $$\begin{array}{l} M=\max(\left|x_{b}-x_{i}\right|,\left|y_{b}-y_{i}\right|,\left|z_{b}-z_{i}\right|)\\ h\left(\alpha \right)=\left\{\begin{array}{l} \alpha ,\alpha =\pm 1\\ 0,-1<\alpha <1 \end{array}\right. \end{array}$$

This is the final step of the source localization. Then, to solve the origin time $t_{0}$, we use the following objective function G:

(9) $$G=\sum_{i=1}^{N-1}\sum_{j=i+1}^{N}{\left|t_{i}^{obs}-T_{ip}^{}-t_{0}\right|}^{m},\;\left(m=1\right).$$

We use PSO (see Appendix A for the pseudocode) to solve Equation (9) and obtain accurate source coordinates and the origin time.

## 3. Results and Discussions

In this section, we first verify the performance of proposed method through synthetic tests and then apply our proposed method to real data.

### 3.1. Synthetic Tests

In this section, we first build a simple tunnel model and then test the accuracy and efficiency of the HVM-based approach with the synthetic microseismic data. Finally, the noise immunity of the HVM-based approach is analyzed by adding the arrival time error, and the SVM-based approach is compared to the HVM-based approach. 

#### 3.1.1. Establishment of the Tunnel Engineering Model

**Figure 2:**
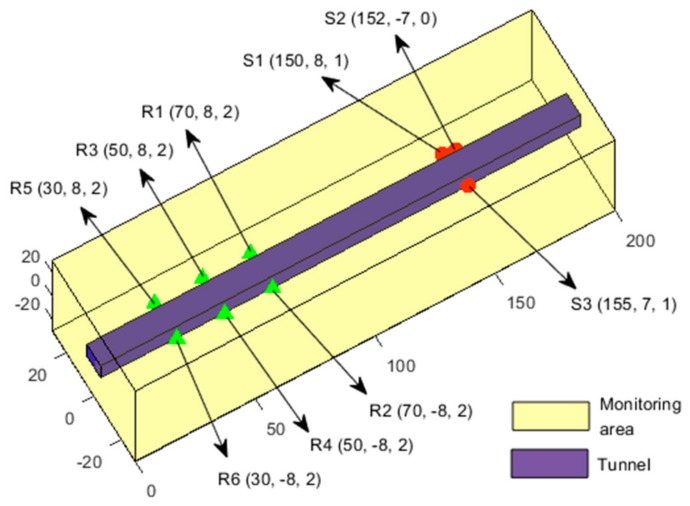
Monitoring model of tunnel engineering.

As shown in Figure 2, the microseismic monitoring area range was a cube, along x coordinates from 0 m to 200 m, y coordinates from −30 m to 30 m, and z coordinates from −30 m to 30 m. A rectangular section of the tunnel, perpendicular to the x direction, was 5 m × 5 m. A total of six receivers were arranged on both sides of the tunnel, which are represented by the green triangles and denoted as R1, R2,..., R6. The three designed seismic sources, represented using red circles, are denoted S1, S2, and S3. The specific parameters of the receivers and seismic sources are shown in Table 1. The wave velocity in the monitoring area was 5000 m/s. However, since the medium in the tunnel after excavation was air, and the energy of the waves in air is greatly attenuated, the propagation velocity in the air was much lower than the propagation velocity in the rock. Therefore, the velocity of the empty area after the tunnel excavation was set to 340 m/s.

**Figure 3:**
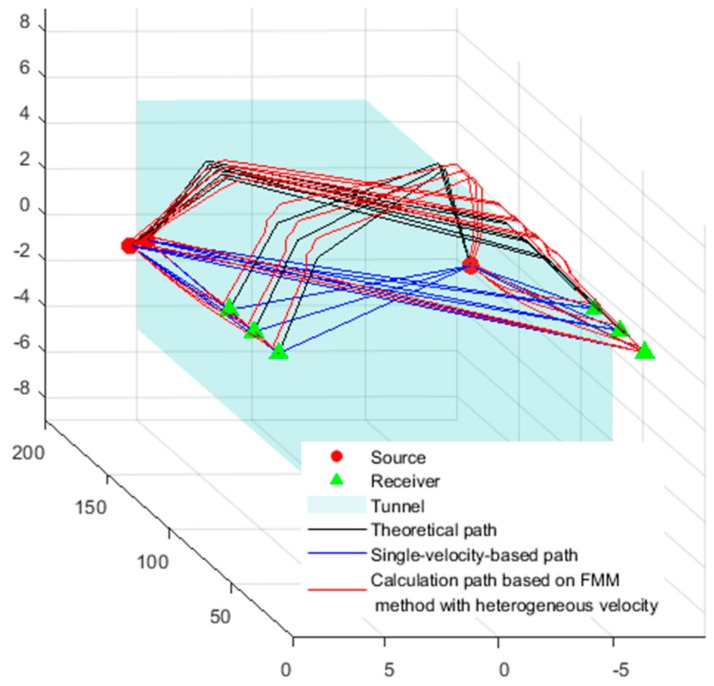
Comparison of the wave propagation trajectories under different velocity models. FMM: fast marching method.

The propagation of waves follow the principle of minimum travel time. As the wave travels slowly in the tunnel after excavation, it bypasses the empty area and travels in the rock. In this example, the rock is homogeneous, and the wave velocity is the same throughout the rock; therefore, the minimum path from the source to the receiver is the shortest path. According to the knowledge of spatial analytic geometry, we can obtain the minimum travel path, namely, the theoretical path, which is represented by a solid black line in Figure 3. 

**Figure 4:**
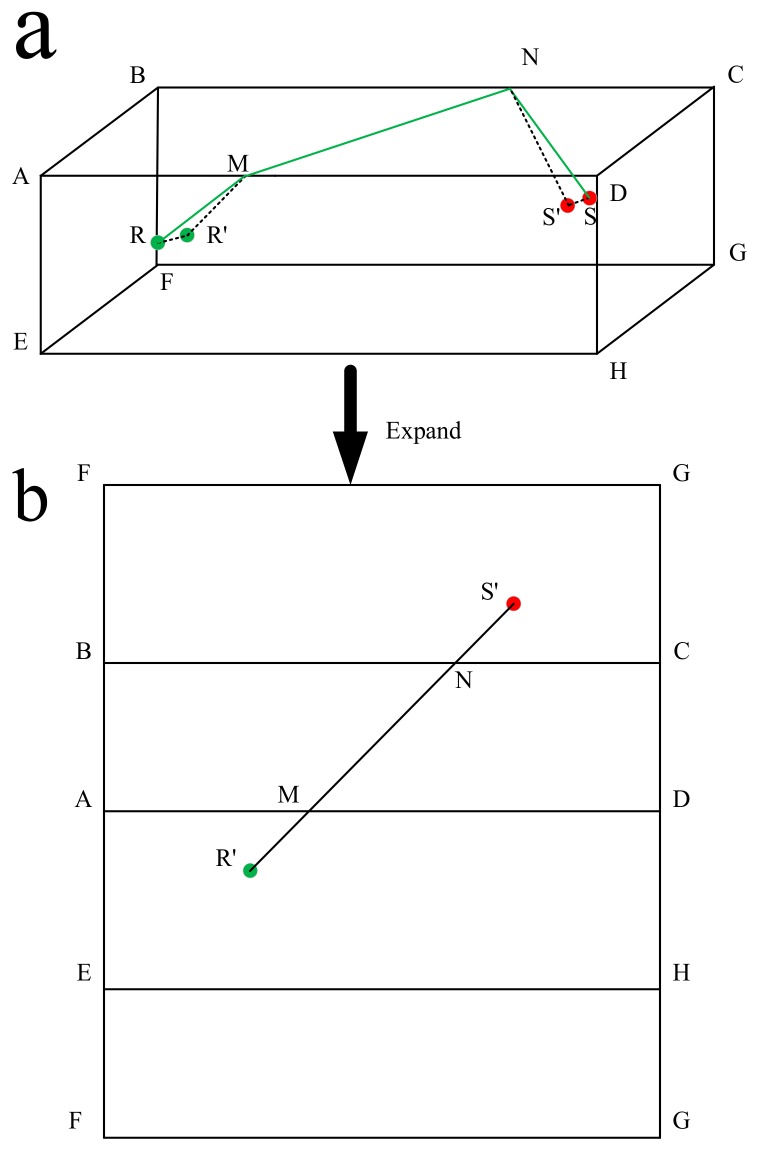
Schematic diagram of the calculation of the theoretical path: (**a**) the tunnel model, and (**b**) expanded side view of the tunnel model.

The specific calculation method of the theoretical path is as follows. Figure 4a is the tunnel model. The faces ADHE and BFGC are the two sides of the tunnel. R is the receiver that is very close to the face ADHE, and S is the source near the face BFGC. R′ and S′ are the vertical projections of R and S on the faces ADHE and BFGC, respectively. We first find the shortest distance from R′ to S′. We expand the tunnel model vertically to obtain Figure 4b. According to the principle that the line segment between two points is the shortest, we obtain the shortest path from S′ to R′, which intersects the line segments AD and BC at points M and N. We then utilize plane DNMR to obtain the shortest path from S to R (green line in Figure 4a). According to the theoretical path, the theoretical arrival time of each source to each receiver can be calculated, as shown in Table 2.

#### 3.1.2. MEL Based on an HVM

Based on the abovementioned tunnel engineering model, we used an HVM to locate the three sources. First, we meshed the monitoring area with a grid size of 0.5 m × 0.5 m × 0.5 m, thus obtaining 400 × 120 × 120 unit blocks. The tunnel geometry model was used as a constraint to assign velocity values to the unit block. The velocity of the unit block inside the tunnel was 340 m/s, and that of the unit block outside the tunnel was 5000 m/s.

BL was carried out using the theoretical arrival time in Table 2 as input parameters. Each block was a potential source, and the residual arrival time of each block was calculated according to Equation (5). The theoretical travel time for the waves between the sources and receivers was calculated using the FMM, as shown in Figure 4. The red lines in the figure represent the calculated path from the sources to the receivers. We used the grid search method to assign the block with the minimum value of Equation (5) as the target block, and the center coordinates of the target block represent the BL results, as shown in Table 3.

**Figure 5:**
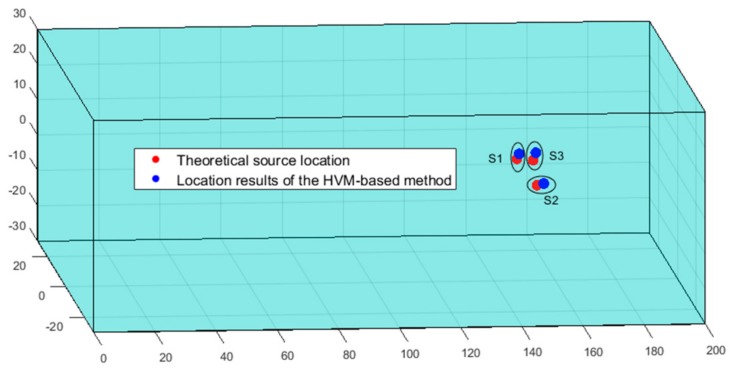
The results of the heterogeneous velocity model (HVM)-based method and theoretical source locations.

**Figure 6:**
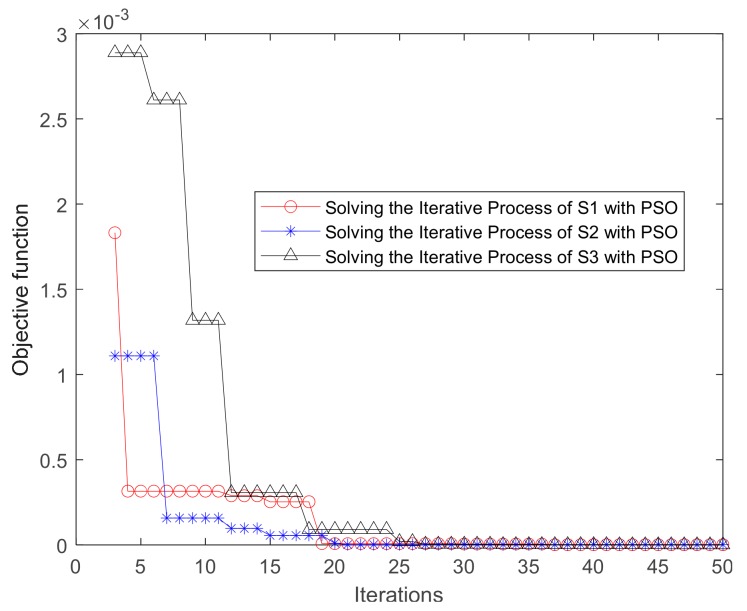
Iterative particle swarm optimization (PSO) process.

AL was then performed in the target block according to Equations (6)–(9). The minimum value of Equation (9) was found using PSO, and the AL results are shown in Table 3. Figure 5 shows that the AL results of the three sources were very close to the theoretical position. The PSO iteration parameters were as follows: The maximum number of iterations was 2000, and the number of seeds was 80. The acceleration parameters of the algorithm were 2 and 2, which affected the local and global optimal values, respectively. The weighted values for the initial and convergence moments were 0.9 and 0.4, respectively. The threshold of the termination algorithm was 1 × 10^−25^, and when the minimum value of the target function was less than this value, the algorithm stopped. The change in the value of the objective function in the PSO iteration is shown in Figure 6, and the three sources converged after 25 iterations.

A description of the computational efficiency is as follows. First, we used the FMM to calculate the travel time of the waves from each receiver to all grids and saved these travel times in a database. As long as the receiver position and velocity model were not changed, each subsequent microseismic event was located via the travel time database, such that the FMM solution needed to be calculated only once. In this paper, the FMM code was based on C++ programming, and the other code was based on MATLAB programming. In this case, it took 51 s to construct the travel time database using the FMM. The BL and AL computation times for the three events are shown in Table 3. After obtaining the travel time database, locating an event took approximately 17 s. All the above programs ran on a 3.6 GHz Intel Core i9-9900k CPU.

**Figure 7:**
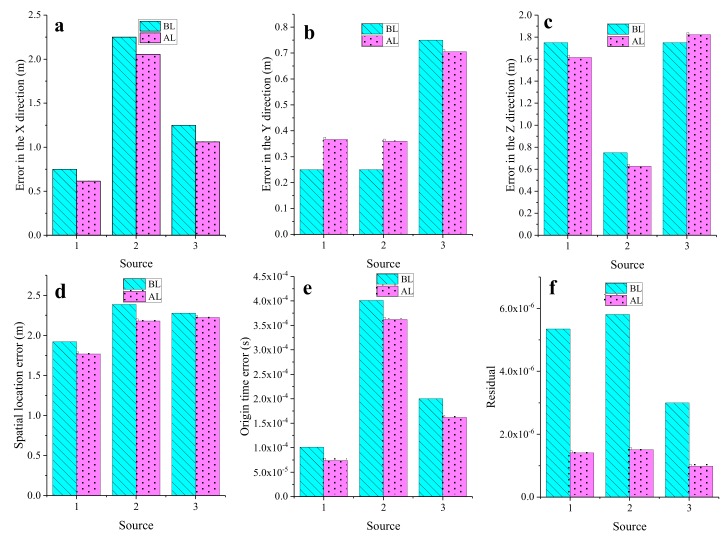
The location errors of AL and BL: (**a**) error in the X direction, (**b**) error in the Y direction, (**c**) error in the Z direction, (**d**) spatial location error, (**e**) origin time error, and (**f**) residual.

As shown in Figure 7, we compared the BL results of the three sources with the AL results. The teal columns in Figure 7 represent the errors of the BL results, and the pink columns represent the errors of the AL results. The location results include the X, Y, and Z coordinate errors, spatial error, origin time error, and minimum value of the target function. The AL errors of S1 and S2 were larger than the BL errors in the Y direction, and the AL errors of S3 were larger than the BL errors in the *Z* direction. We calculated the spatial location error using Equation (10):

(10) $$\Delta SR=\sqrt{\Delta XR^{2}+\Delta YR^{2}+\Delta ZR^{2}},$$

where

(11) $$\left\{\begin{array}{l} \Delta XR=X^{c}-X^{t}\\ \Delta YR=Y^{c}-Y^{t}\\ \Delta ZR=Z^{c}-Z^{t} \end{array}\right..$$

ΔSR represents the spatial location error. ΔXR, ΔYR, and ΔZR represent the location errors of the X, Y, and Z coordinates, respectively. $X^{c}$, $Y^{c}$, and $Z^{c}$ represent the location results of the X, Y, and Z coordinates, respectively. $X^{t}$, $Z^{t}$, and $Z^{t}$ represent the theoretical values of the X, Y, and Z coordinates, respectively.

However, the AL results of the three sources were clearly smaller than the BL results in terms of the spatial error, which indicates that the AL results were closer to the theoretical position. In addition, in terms of the error of the origin time and the minimum value of the objective function, the AL results were also better than the BL results.

#### 3.1.3. Comparison and Analysis

Due to the influence of noise in the tunnel, there was a certain error in the arrival picking in actual projects. To further test the practicality of the HVM-based method, we added a certain noise in the theoretical arrival time (see Table 2). We designed three contrasting experiments. First, the theoretical arrival time was used as the location input parameter, and an SVM was adopted to locate the three sources, which was expressed using the SVM. Second, the noisy arrival time was used as the location input parameter, and an HVM was adopted to locate the three sources, which was represented using the HVM(N), where N is stand for noisy arrival time. Third, the noisy arrival time is used as the location input parameter, and an SVM was adopted to locate the three sources, which is represented using the SVM(N). The location results of the three experiments are shown in Table 4, which clearly shows that the origin time obtained by these three experiments was very close to the theoretical origin time. However, the location results of the SVM and SVM(N) were significantly different from those of the HVM(N) in the Y direction.

**Figure 8:**
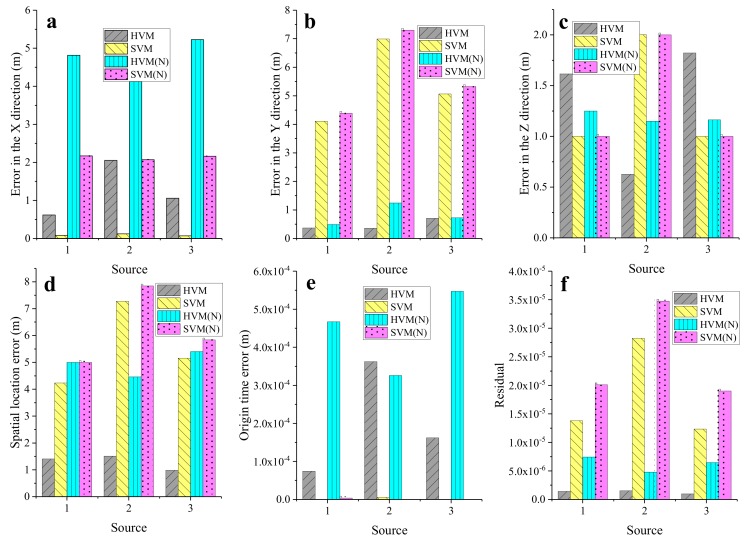
Error of location results of the four experiments: (**a**) error in the X direction, (**b**) error in the Y direction, (**c**) error in the Z direction, (**d**) spatial location error, (**e**) origin time error, and (**f**) residual.

We used these three experiments to compare the errors of the location results of the HVM and SVM in detail, as shown in Figure 8. The origin time error of these four experiments was within 0.6 ms, and the accuracy was very high, which was not used as a criterion. In the case where no noise was added, the location error of the SVM of the three sources in the X direction was smaller than that of the HVM, but the location error of the HVM in the Y direction was smaller than that in the SVM. The location errors of the SVM in the Z direction of S1 and S3 were smaller than those of the HVM, but the opposite was true for S2. Considering the location errors in the X, Y, and Z directions, it was impossible to determine which method had a higher location accuracy. The spatial location error was a comprehensive error in the integrated X, Y, and Z directions. Therefore, the spatial location error calculated using Equation (10) was used as the criterion.

Figure 8 clearly shows that the spatial location error of the HVM was smaller than the spatial location error of the SVM. For the average spatial location errors of the three sources, HVM (2.06 m) < SVM (5.51 m). In addition, from the minimum value of the objective function, the value of the HVM-based MEL was smaller. In the case with the added noise, the spatial location error and the residual of the HVM-based method were smaller than those of the SVM-based method. Regarding the average spatial location error of the three sources, HVM (4.95 m) < SVM (6.23 m). In summary, it can be concluded that the location accuracy of the HVM was higher than that of the SVM. Below, we use the proposed method to analyze real data.

### 3.2. Application to Real Data

**Figure 9:**
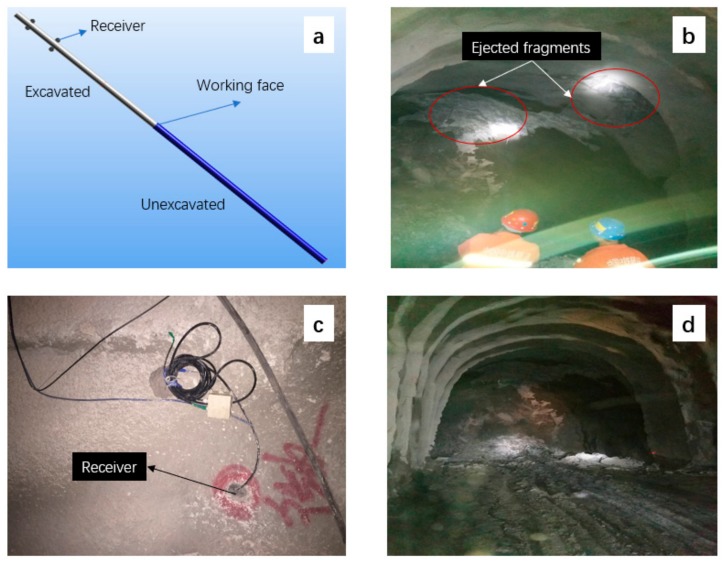
Tunnel engineering conditions: (**a**) receiver layout and progress of tunnel excavation, (**b**) ejected fragments on the top of the working face, (**c**) receiver position indicated by red paint, and (**d**) working face of the project.

The Qinling No. 4 tunnel of the Yinhanjiwei project is located south of the Qin Mountains in southern Shaanxi Province, China. The length of this shaft is 5820 m, with a section 6.5 m high and 6.7 m wide. The maximum slope is 11.96%, and the maximum depth is 1600 m. Drilling and blasting are used in this project, resulting in frequent rockbursts that may both damage infrastructure and injure people. To provide safety guidance, a microseismic system was used for 24 h of continuous monitoring. Four accelerometers with a sensitivity of 10 V/g were embedded along two sides of the tunnel. The spatial arrangement of the receiver is shown in Figure 9a. The sampling frequency was set to 10 kHz.

The working face of the actual project is shown in Figure 9d. Due to the poor lighting inside the tunnel, to distinguish the microseismic hole, the sensor installation position was marked with red paint for visibility, as shown in Figure 9c. Some ejected fragments were found at the top of the working face on 15 June 2017, as shown in Figure 9b. According to engineering experience, these fragments formed due to the destruction of the roof rock mass caused by the excavation of the tunnel. Below, we verify this through microseismic monitoring.

**Figure 10:**
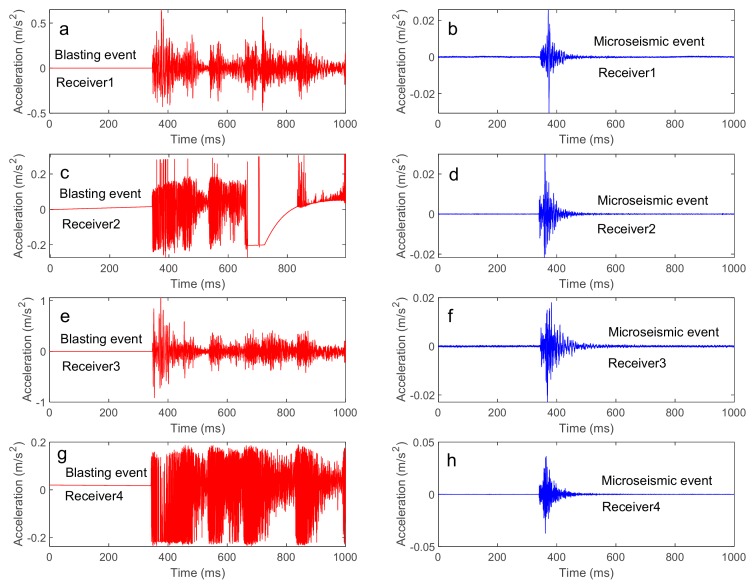
Waveforms of the blasting event and waveforms of the microseismic event: (**a**,**c**,**e**,**g**) are the waveforms collected by Receiver1, Receiver2, Receiver3, Receiver4 for the blasting event. (**b**,**d**,**f**,**h**) are the waveforms collected by Receiver1, Receiver2, Receiver3, Receiver4 for the microseismic event.

During the tunneling process, the microseismic monitoring system monitored a large number of events. The microseismic event and the blasting event could be clearly distinguished by the waveform, as shown in Figure 10. We selected 51 events with better waveforms during the period from 6 June 2017 to 13 June 2017, including 7 blast events and 44 microseismic events. We verified the location effect in two ways: (1) due to the known blasting position of the working face, we verified the location effect based on 7 blasting events; and (2) we verified the location effect based on the spatial relationship between the location results of 44 microseismic events and the working surface.

**Figure 11:**
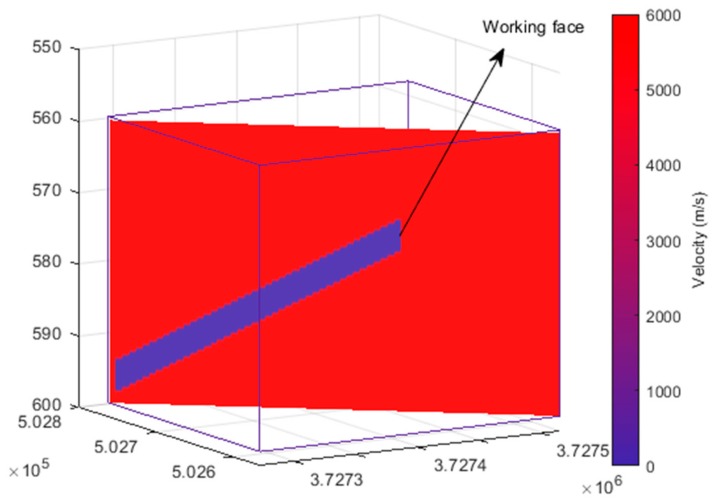
3D HVM: Slices of the velocity model within the 3D monitoring volume.

We used the HVM to locate these 51 events. The monitoring area covered the x coordinates from 3,727,271 m to 3,727,516 m, y coordinates from 502,564 m to 502,761 m, and z coordinates from 558 m to 598 m. The block size was 0.5 m × 0.5 m × 0.5 m, so there were a total of 490 × 394 × 80 unit blocks. There are many ways to calculate the propagation velocity of waves in rock masses. For example, Wang et al. optimized the seismic wave velocity in the deep mining area of a coal mine by using a combination method, residual error optimization method, location error optimization method, location residual optimization method, and combined inversion method [33]. The acquisition of the velocity model was not the focus of this paper. In this application, the wave propagated at a velocity of approximately 6000 m/s in the rock mass. Therefore, we set the velocity of the unexcavated rock mass to 6000 m/s and the velocity of the empty area after excavation to 340 m/s, as shown in Figure 11. The coordinates of the receivers and the arrival time of the seven blasting events are shown in Table 5. The arrival time of the 44 microseismic events is shown in Appendix B.

**Figure 12:**
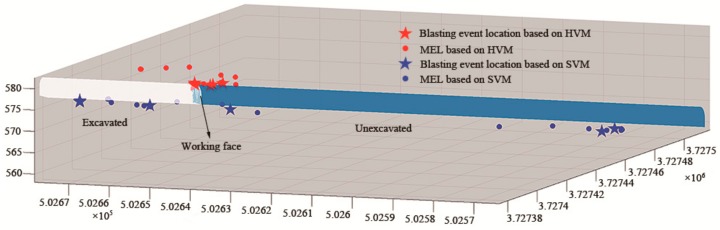
Spatial distribution of the microseismic event location (MEL) results based on the HVM and SVM.

The location results are shown in Figure 12. In this figure, red indicates the location result of the HVM, and blue indicates the location result of the SVM; five-pointed stars indicate the blast events, and circles indicate the microseismic events. The results of the seven blasting events are shown in Table 6. The results of the 44 microseismic events are shown in Appendix C.

First, we analyzed the results of the HVM. From the location results of the blasting events, it can be clearly seen that the seven blasting events occurred in the vicinity of the working face, which was consistent with the actual engineering excavation. The location results of the microseismic events showed that 44 microseismic events were concentrated around the blasting events. The blasting of the tunnel face caused damage to the surrounding rock mass. The microseismic signal from the rock mass damage could be received by the sensor; therefore, in theory, most of the events occurred near the working face. The location results of the 44 microseismic events were consistent with this theoretical derivation.

Then, we analyzed the location results of the SVM. As shown in Figure 12, the location results of the blasting events suggested that the SVM results were very scattered. Four of the blasting events were located behind the propulsion direction of the working face, and two blasting events were located approximately 85 m in front of the propulsion direction of the working face. From the location results of the microseismic events, the spatial distribution of the 44 microseismic events was consistent with that of the seven blasting events. The spatial distribution was very scattered, irregular, and not concentrated near the working surface.

In summary, we can conclude that the HVM had a high location accuracy and good effect, with a location accuracy that was much higher than that of the SVM.

What caused the location results of the SVM-based method to be so poor? We compared these results with the HVM-based MEL and found that the velocity model error decreased the location accuracy. After excavation, the tunnel was filled with air, and the propagation of the microseismic signal was greatly affected. The wave did not pass directly through the empty zone to the receiver, but rather bypassed the empty zone and reached the receiver by travelling through the unexcavated rock mass. Therefore, in the MEL in the tunnel, we should have fully considered the impact of the empty zone and used the HVM for location.

The HVM-based method proposed in this paper had a high precision and was suitable for MEL during tunnel excavation. The location accuracy of the HVM varied with the size of the mesh. The larger the mesh size was, the lower the accuracy. Conversely, the smaller the mesh size was, the higher the accuracy. However, the smaller the grid was, the lower the computational efficiency. Therefore, in practical engineering applications, we should consider both accuracy and efficiency when determining the appropriate grid size.

## 4. Conclusions

We adopted a grid-based modeling method that can express arbitrary velocity models and establish a 3D HVM in the microseismic monitoring area of an excavated tunnel. For the velocity model the tunnel, we fully considered the influence of the empty area on the location result and set the velocity in the tunnel after excavation to 340 m/s. The approximate range of the source was determined using BL, and then the exact position of the source was determined using the AL.

The synthetic tests showed that the location accuracy of the proposed HVM-based method was higher than that of the traditional SVM-based method and that the proposed method had certain anti-interference characteristics. The average spatial location error of the HVM was less than that of the SVM: HVM (2.06 m) < SVM (5.51 m) without adding noise. With added noise, the location accuracy of the HVM-based method was also higher than that of the SVM-based method: HVM (4.95 m) < SVM (6.23 m). Finally, the HVM-based method was applied to the monitoring of the Qinling No. 4 tunnel of the Yinhanjiwei project. The results showed that the event locations of the HVM were concentrated near the working surface, which was in line with observations made during engineering practice. However, the event distribution of the SVM-based method was very scattered and irregular. Therefore, the empty area created by the excavation had a great influence on the microseismic event location around the tunnel. The HVM improved the location accuracy of microseisms around the tunnel and has practical research significance.

## Figures and Tables

**Table 1:** Receiver coordinates and theoretical source parameters.

Parameters	R1	R2	R3	R4	R5	R6	S1	S2	S3
X (m)	70	70	50	50	30	30	150	152	155
Y (m)	8	−8	8	−8	8	−8	8	−7	7
Z (m)	2	2	2	2	2	2	1	0	1
Origin Time (ms)	-	-	-	-	-	-	800	800	800

**Table 2:** Theoretical arrival time and noisy arrival time of each receiver.

Source	Arrival Time (ms)	R1	R2	R3	R4	R5	R6
S1	Theoretical arrival time	816.001	816.478	820.001	820.385	824.001	824.322
	Noisy arrival time	816.446	816.923	820.448	820.832	824.450	824.771
S2	Theoretical arrival time	816.940	816.406	820.839	820.405	824.769	824.404
	Noisy arrival time	817.386	816.851	821.287	820.852	825.219	824.854
S3	Theoretical arrival time	817.002	817.449	821.002	821.366	825.002	825.309
	Noisy arrival time	817.448	817.895	821.450	821.814	825.452	825.759

**Table 3:** Block localization (BL) results and accurate location (AL) results.

Location	Source	X (m)	Y (m)	Z (m)	T (ms)	Time Elapsed (s)	Residual (×10^−6^)
BL	S1	150.750	7.750	2.750	799.899	15.39	5.346
	S2	154.250	−7.250	0.750	799.599	14.49	5.805
	S3	156.250	7.750	2.750	799.800	14.75	2.997
AL	S1	150.615	7.633	2.615	799.926	1.37	1.405
	S2	154.054	−7.360	0.627	799.638	1.22	1.509
	S3	156.060	7.705	2.822	799.838	1.29	0.982

**Table 4:** Location results of the three comparative experiments. SVM: single-velocity model, HVM: heterogeneous velocity model, (N): Noisy arrival time.

Location	Source	X (m)	Y (m)	Z (m)	T (ms)	Residual (10^−6^)
SVM	S1	149.920	12.111	2.000	800.000	13.801
	S2	151.877	−13.993	2.003	799.994	28.234
	S3	154.931	12.064	2.000	800.000	12.332
HVM(N)	S1	154.815	7.514	2.250	799.533	7.440
	S2	156.125	−8.243	1.149	799.674	4.793
	S3	160.226	7.724	2.164	799.453	6.443
SVM(N)	S1	152.168	12.388	2.000	799.996	20.112
	S2	154.071	−14.301	2.000	800.000	34.751
	S3	157.161	12.326	2.000	800.000	19.003

**Table 5:** Receiver coordinates and arrival time of the seven blasting events.

	Receivers			Arrival Time of the Blast Events						
	X (m)	Y (m)	Z (m)	1	2	3	4	5	6	7
1	3,727,396.00	502,657.70	574.40	387.50	387.64	387.19	385.30	347.04	346.30	346.80
2	3,727,500.85	502,584.39	558.87	383.80	383.40	383.08	381.59	342.90	342.70	342.90
3	3,727,413.24	502,644.24	571.95	388.10	387.73	387.21	385.81	347.10	346.70	347.00
4	3,727,510.86	502,584.86	558.27	383.70	383.38	382.99	381.60	342.69	342.40	342.60

**Table 6:** Location results of the SVM and HVM for the seven blasting events.

	SVM			HVM		
	X (m)	Y (m)	Z (m)	X (m)	Y (m)	Z (m)
1	3,727,396.00	502,657.70	574.40	3,727,410.00	502,648.10	577.73
2	3,727,500.85	502,584.39	558.87	3,727,414.74	502,646.63	577.79
3	3,727,413.24	502,644.24	571.95	3,727,411.15	502,647.77	577.84
4	3,727,510.86	502,584.86	558.27	3,727,409.86	502,651.83	578.22
5	3,727,380.50	502,669.50	576.64	3,727,406.76	502,650.55	578.14
6	3,727,380.50	502,669.50	576.58	3,727,406.90	502,650.68	578.24
7	3,727,380.50	502,669.50	576.86	3,727,406.60	502,650.59	578.37

## Appendix A. The PSO Pseudocode

**Procedure PSO**

 **For** each particle i

  Initialize velocity Vi and position Xi for particle i

  Evaluate particle i and set pBesti=Xi

 **End for**

 $gBest=\min\left\{pBesti\right\}$

 **While** not stop

  **For** i=1 to N

   Update the velocity and position of particle i

   Evaluate particle i

   **If** $fit\left(Xi\right)<fit\left(pBesti\right)$

    pBesti=Xi

   **If** $fit\left(pBesti\right)<fit\left(gbest\right)$

    gBest=pBesti

  **End for**

 **End while**

 Print

**End procedure**

## Appendix B

**Table A1:** Arrival Times of the 44 Microseismic Events.

Events	R1	R2	R3	R4
1	919.9	915.2	920.1	915
2	382.5	378.8	383.3	378.6
3	384	378.7	384.4	379.5
4	384.7	380.6	384.7	380.2
5	384.9	380.9	384.9	380.6
6	382.2	378.4	382.3	377.9
7	383.9	380.1	383.9	379.716
8	386.3	382.5	386.7	382.2
9	383.3	379.6	383.3	378.6
10	385.801	381.7	386.2	381.9
11	384.686	380.659	384.686	380.506
12	383.87	380.1	383.9	380.021
13	378.67	374.7	379.614	374.707
14	386.98	382.8	386.98	382.7
15	381.286	377.3	381.9	377.3
16	385.385	381.07	385.457	381.106
17	382.1	377.7	382.5	377.8
18	382.4	378.5	382.4	377.9
19	386.3	382.3	386.8	382.2
20	661.4	657.4	661.6	657.3
21	385.228	381.3	385.5	381.196
22	259.184	255.195	259.7	255.3
23	393.9	390.072	393.9	389.832
24	399.147	395.529	399.207	395.096
25	385.4	381.502	385.8	381.502
26	384.447	380.5	384.712	380.024
27	385.385	381.4	385.6	381.292
28	384.297	380.4	384.808	380.4
29	384.5	380.7	384.8	380.5
30	383.413	379.4	383.9	379.4
31	384.627	380.7	384.928	380.5
32	385.793	381.7	385.871	381.587
33	384.3	380.4	384.3	380.072
34	345.2	341.3	345.4	341.1
35	808.237	804.195	808.365	804.159
36	345.7	341.7	345.7	341.388
37	345.096	341.1	345.1	340.799
38	346.01	341.8	346	341.394
39	344.519	340.7	345.1	340.7
40	344.5	340.3	344.519	339.808
41	344	340.1	344.4	339.8
42	347.296	343.5	347.296	342.891
43	343.69	339.567	343.894	339.591
44	343.835	339.5	344	339.5

## Appendix C

**Table A2:** Location Results Based on the HVM and SVM for the 44 Microseismic Events.

Events	HVM-Based MEL				SVM-Based MEL			
	X (m)	Y (m)	Z (m)	T (ms)	X (m)	Y (m)	Z (m)	T (ms)
1	3,727,380.500	502,669.500	576.597	907.559	3,727,406.989	502,650.848	578.219	901.792
2	3,727,380.500	502,669.500	576.920	370.677	3,727,406.770	502,650.802	578.311	365.457
3	3,727,401.819	502,670.341	574.598	368.804	3,727,403.100	502,662.748	581.817	368.166
4	3,727,380.500	502,669.500	576.876	372.807	3,727,406.630	502,650.790	578.366	367.431
5	3,727,380.500	502,669.500	576.678	372.762	3,727,406.706	502,650.831	578.182	367.579
6	3,727,380.500	502,669.500	576.553	370.540	3,727,406.747	502,650.753	578.303	364.818
7	3,727,380.500	502,669.500	576.839	371.992	3,727,406.607	502,650.800	578.438	366.759
8	3,727,380.500	502,669.500	576.903	374.620	3,727,406.780	502,650.912	578.377	369.170
9	3,727,380.500	502,669.500	576.751	371.610	3,727,406.795	502,650.858	578.371	365.964
10	3,727,401.729	502,670.161	574.669	371.496	3,727,403.213	502,662.631	581.795	370.621
11	3,727,380.500	502,669.500	576.836	372.796	3,727,406.725	502,650.771	578.251	367.459
12	3,727,419.144	502,639.688	570.709	363.764	3,727,409.143	502,649.347	578.229	366.418
13	3,727,507.648	502,585.637	558.244	341.168	3,727,410.062	502,651.214	578.159	361.012
14	3,727,395.884	502,657.735	574.315	371.613	3,727,410.371	502,648.107	577.763	368.769
15	3,727,509.809	502,582.784	558.456	343.631	3,727,415.177	502,647.206	577.935	362.422
16	3,727,509.906	502,590.859	558.103	348.298	3,727,417.145	502,647.976	579.372	366.462
17	3,727,429.430	502,652.117	570.869	361.477	3,727,413.271	502,654.367	581.427	364.124
18	3,727,380.500	502,669.500	576.901	370.436	3,727,406.678	502,650.836	578.350	365.315
19	3,727,395.884	502,657.643	574.125	371.063	3,727,410.137	502,648.271	577.823	368.450
20	3,727,395.746	502,657.671	574.174	646.154	3,727,410.269	502,648.174	577.904	643.346
21	3,727,394.193	502,658.616	574.500	370.515	3,727,407.272	502,650.267	578.340	367.602
22	3,727,418.245	502,659.218	572.485	241.128	3,727,407.898	502,658.135	581.723	242.646
23	3,727,380.500	502,669.500	576.733	382.167	3,727,406.943	502,650.774	578.142	376.792
24	3,727,380.500	502,669.500	576.894	387.471	3,727,406.621	502,650.718	578.379	382.015
25	3,727,509.871	502,582.642	558.261	347.563	3,727,415.340	502,647.420	577.704	366.835
26	3,727,380.500	502,669.500	576.689	372.655	3,727,406.873	502,650.518	578.213	367.322
27	3,727,392.667	502,659.845	574.823	370.930	3,727,406.602	502,650.814	578.352	368.155
28	3,727,509.888	502,582.750	558.174	346.498	3,727,415.212	502,647.107	577.701	365.660
29	3,727,380.500	502,669.500	576.587	372.700	3,727,406.690	502,650.729	578.203	367.261
30	3,727,509.843	502,582.807	558.348	345.802	3,727,415.441	502,647.292	577.890	364.662
31	3,727,380.500	502,669.500	576.595	372.900	3,727,406.569	502,650.535	578.140	367.388
32	3,727,387.417	502,664.235	575.809	372.112	3,727,406.820	502,650.688	578.326	368.236
33	3,727,380.500	502,669.500	576.715	372.700	3,727,406.904	502,650.709	578.245	366.871
34	3,727,380.500	502,669.500	576.664	333.497	3,727,406.900	502,650.558	578.270	328.166
35	3,727,475.707	502,600.572	562.301	776.971	3,727,417.280	502,644.365	577.278	788.635
36	3,727,380.500	502,669.500	576.745	333.622	3,727,406.634	502,650.664	578.426	328.129
37	3,727,380.500	502,669.500	576.613	333.253	3,727,406.711	502,650.623	578.221	328.043
38	3,727,380.500	502,669.500	576.727	334.082	3,727,406.675	502,650.845	578.338	328.459
39	3,727,509.604	502,582.783	558.146	306.864	3,727,415.208	502,647.404	577.717	325.694
40	3,727,380.500	502,669.500	576.557	332.552	3,727,406.690	502,650.659	578.396	327.434
41	3,727,380.500	502,669.500	576.554	332.386	3,727,406.638	502,650.546	578.150	326.889
42	3,727,380.500	502,669.500	576.596	335.377	3,727,406.742	502,650.814	578.209	330.118
43	3,727,498.615	502,595.706	559.913	308.884	3,727,419.631	502,645.318	578.732	324.141
44	3,727,509.760	502,582.734	558.201	305.788	3,727,415.272	502,647.267	577.870	324.529
